# Blood Analysis of Laboratory *Macaca mulatta* Used for Neuroscience Research: Investigation of Long-Term and Cumulative Effects of Implants, Fluid Control, and Laboratory Procedures

**DOI:** 10.1523/ENEURO.0284-21.2021

**Published:** 2021-10-19

**Authors:** Detlef Wegener, Dan Qi Priscilla Oh (胡箪棋), Herbert Lukaß, Michael Böer, Andreas K. Kreiter

**Affiliations:** 1 Brain Research Institute, Center for Cognitive Science, University of Bremen, 28334 Bremen, Germany; 2 Department of Biology, University of Bremen, 28334 Bremen, Germany; 3 Institute of Terrestrial and Aquatic Wildlife Research, University of Veterinary Medicine, 30559 Hannover, Germany

**Keywords:** cumulative effects, long-term effects, neuroscientific procedures, non-human primate, severity

## Abstract

The nonhuman primate (NHP) constitutes an extraordinarily important model in neuroscience research for understanding the neuronal underpinnings of perceptual, motor, cognitive, and executive functions of the primate brain, and to study the physiological causes, effects, and potential treatments of brain disorders. Because of their cognitive capabilities, NHPs receive special attention in animal welfare regulations around the world, and their well-being is a benchmark for the evaluation, monitoring, and refinement of experimental procedures. As a consequence, many typical neuroscientific procedures are considered only mildly severe by animal welfare boards. There is, however, an ongoing debate about possible long-term and cumulative effects. Because of a lack of longitudinal data, it is unclear whether mildly severe procedures may cause more significant harm on the long-term, and to what extent they may impact animal well-being and healthiness over time. We here make use of a database of blood samples drawn over a period of 15 years from 39 rhesus monkeys (*Macaca mulatta*) to address the issue of long-term, cumulative effects of neuroscientific procedures. A careful analysis of indicative primate blood markers for chronic inflammation, hydration status, and stress levels, their comparison to baseline values from both the same animals and the literature, and evaluation of additional hematologic, physiological, and behavioral parameters did not provide support for the notion of long-term, cumulative effects on the monkeys’ healthiness and well-being. The results may serve the community as a reference for the severity assessment of neuroscientific experiments involving NHPs.

## Significance Statement

Whether and to what extent standard nonhuman primate (NHP) neuroscientific procedures would cause cumulative harm, either because of combining different methods or over time, is an important concern addressed by both legislative guidelines and local authorities. We have analyzed a database of blood samples drawn over a period of 15 years with regard to cranial implants, fluid protocols, and laboratory procedures, supplemented by veterinarian records on physiological and behavioral attributes. A thorough investigation of the data and their comparison to baseline values from the same animals, taken before start of laboratory work, does not support the notion of cumulative effects. The results show that monkeys may well adapt to laboratory procedures and successfully manage their health and well-being as during baseline periods.

## Introduction

Because of their genomic and physiological similarity to humans, non-human primates (NHPs) constitute an indispensable laboratory model for various fields of biomedical research, including brain research, neurology, hematology, and immunology (Anderson, 2008; Gardner and Luciw, 2008; Evans and Silvestri, 2013; Roelfsema and Treue, 2014; Verdier et al., 2015), with research on SARS-CoV2 (Chandrashekar et al., 2020; Deng et al., 2020; Munster et al., 2020) and preclinical testing of vaccine candidates (Corbett et al., 2020; Mercado et al., 2020; van Doremalen et al., 2020; Vogel et al., 2021) to fight the COVID-19 pandemic as recent examples. In systems neuroscience, NHP research led to the establishment of theories on complex neuronal interactions required to understand and eventually treat brain diseases and mental disorders (Uhlhaas and Singer, 2006; Siegel et al., 2012; Fries, 2015; Kreiter, 2020) and to the discovery of the mirror neuron system (Rizzolatti and Craighero, 2004), to name two key areas of research. Macaque monkeys represent the long-standing model for understanding the human brain in health and disease, because of the degree of similarity in brain anatomy and connectivity (Orban et al., 2004; Rauschecker and Scott, 2009; Petrides et al., 2012; Thiebaut de Schotten et al., 2012), motor actions (Munoz and Everling, 2004; Rizzolatti and Rozzi, 2016; Castiello and Dadda, 2019), and perceptual, cognitive, and social abilities (Deaner and Platt, 2003; Kastner et al., 2008; Hosokawa and Watanabe, 2012; Rajalingham et al., 2015; Hanks and Summerfield, 2017; Gray and Barnes, 2019; Roberts and Clarke, 2019), recently supplemented by the common marmoset (*Callithrix jacchus*) to facilitate genetic engineering studies (Tokuno et al., 2015).

Because of the ethical constrains arising from their cognitive and behavioral capabilities, the work with NHPs requires particularly careful behavioral and laboratory management protocols to ensure their welfare in husbandry and experiment. Besides other measures, this includes regular acquisition of hematologic and biochemical blood parameters as important indicators of an individual’s health and well-being (Bernacky et al., 2002). In NHP systems neuroscience research, regular blood sampling may help to identify issues arising from typical procedures, as, e.g., implantation of a headpost and recording chamber (or other equipment used for signal acquisition), protocols for controlled water or food supply, transfer to and stay in laboratory space, and long periods of data acquisition (both daily and absolute), all of which may exerts an influence on physiological parameters and shows up in hemograms. Careful scientific analysis of such procedures is considered a constant task in the community, and many refinements have been implemented because of the results of systematic investigations (for expert group review, see Prescott et al., 2010). There is, however, a lack of longitudinal studies to clarify whether procedures indicating no or mild severity on the short term may exert cumulative and more severe effects on the long-term. Accordingly, the EU Directive 2010/63 on the use of animals in research particularly emphasizes the “lifetime experience” of laboratory animals and requests assessment of possible cumulative effects related to specific scientific procedures for assignment of severity categories.

We here make use of a blood sample database acquired over a period of 15 years. We address the questions whether and to what extent typical neuroscientific procedures may cause hematologic deviations from baseline, with special emphasis on cumulative, long-term effects. All samples were drawn under largely identical conditions of husbandry and laboratory procedures, and were analyzed by the same biomedical lab to minimize hidden variance. We address whether there would be firstly, chronic inflammatory responses because of cranial implants, secondly, altered kidney values because of controlled fluid supply, and thirdly, stress responses because of laboratory procedures during training and recording periods. All data are compared with baseline values obtained from the same animals before implantation and start of laboratory procedures, and to published reference values. Blood parameter analysis is supplemented by an evaluation of >2000 score sheets on outer appearance and behavior of the monkeys, assessed by an external veterinarian, to address possible changes in general health conditions over the course of time.

The results do not provide statistical evidence of cumulative effects arising from long-term presence of cranial implants, repeated periods of fluid control, or continuing exposure to laboratory procedures, or from the combination of these methods. Given adherence to state-of-the-art husbandry and laboratory methods, they suggest that monkeys may well adapt to experimental settings and effectively manage their health and well-being.

## Materials and Methods

### Animals and husbandry conditions

The database consisted of a total of *N *=* *248 blood samples drawn from 39 male rhesus monkeys over a period of 15 years (2006–2020). All monkeys were bred for scientific purposes and were obtained from the German Primate Center. Monkeys were aged between 2 and 18 years at the time of sampling. They were kept in groups of two or four animals and were housed in large compartments providing indoor and outdoor areas, swings, ropes, climbing opportunities and a manifold of environmental enrichments, hiding areas and sufficient space for vertical flights. Some elderly animals were housed singly in indoor compartments with visual and auditory contact to other animals. Health and well-being were daily monitored by animal caretakers and scientific personnel and by regular veterinary visits (H.L.). All behavioral training on husbandry and laboratory requirements was strictly based on positive reinforcement, including training to enter primate chairs. No collars were used. Husbandry training was performed using fruits as reinforcer, and laboratory training used juice and/or water as reinforcer, depending on the individual monkey’s preferences. Husbandry conditions and procedures, veterinary, laboratory, surgical, and experimental procedures followed the Directive 2010/63, issued by the European Parliament (European Parliament, 2010), and the Regulation for the Welfare of Experimental Animals, issued by the Federal Government of Germany, and were approved and regularly controlled by the local authorities.

#### Cranial implants

Implants in the current study consisted of caps made from bone cement and dental acrylic, as described in detail by Gardiner and Toth (1999). Different from the original protocol, cranial implants were implanted in a bipartite procedure, which allowed for a significant refinement over the traditional protocol. In a first surgery, only a thin layer of bone cement (anchored by medical screws) was laid out and the untrimmed skin was then placed in its original position, covering the cap, and closed by subcutaneous, interrupted sutures. A small opening (∼1 cm in diameter) remained at, or close to, the center of the new implant, typically at the intersection of a Y-cut to open the skin. Because the skin will not attach to the implant, it will slowly retract and eventually insert at the skull very close to the margins of the cap during the next four weeks, approximately. The main refinement is that, before retraction is completed, implant margins are covered by skin tissue and receive full immune protection. This minimizes the risk of infections, prevents undue growth of granulation tissue, and, as a consequence, strongly supports a firm attachment of the skin exactly at the margin of the cap. The typical result is a lasting condition (usually several years) during which routine care required for the cap is reduced to a minimum. After the skin firmly attached to the skull, headholder, connectors and recording chambers were mounted/implanted in a second procedure.

### Data acquisition

Blood samples in the database include blood drawn for yearly routine check, blood drawn when a potential health issue was raised, and blood drawn when an animal underwent surgery. All blood samples were indexed according to the reason of sampling (routine/health issues), status at the time of blood draw (awake/anesthetized), presence of a cranial implant (non-implanted/implanted), and experimental condition (lab/non-lab periods). Samples taken during non-lab periods were further indexed as either drawn shortly (3–10 d) after a lab period or drawn at later times (>10 d). For each sample, the database also held information about the dates of sample draw, monkey birth, and implantation surgery. Combination of indexes and consideration of time information allowed to differentiate suitable subsets of samples to address each specific question in this study.

#### Sampling and anesthesia

Blood samples were drawn from both awake and anesthetized animals. In awake procedures, monkeys entered a horizontal primate chair, in which they sat in a Sphinx-like position and the sample was drawn from its leg by opening the rear side of the chair. If an animal was not trained on the procedure at the time of sampling, it was lightly sedated with a low amount of ketamine/medetomidine. In the event of a surgery, monkeys were given *ad libitum* access to water for at least 48 h. Water bottles were removed either the evening before the surgery (older samples) or about 2 h before surgery. Blood samples were taken in the first 60 min of the surgery. Initial anesthesia was typically performed using a mix of ketamine (4–8 mg/kg body weight) and medetomidine (0.04 mg/kg body weight), supplemented with a low dose of isoflurane (<0.5%). All blood samples in the database were taken from veins. Only samples from non-sedated animals were indexed as “awake.” All other samples were indexed as “anesthetized.”

#### Fluid protocol during baseline, laboratory, and non-laboratory periods

Based on their indexing, all blood samples in the database were assigned to either baseline, laboratory, or non-laboratory time periods. The baseline period is comprising all samples taken before implantation of the cranial cap and start of laboratory training. The laboratory period is comprising all samples drawn after implantation of the cranial cap, during periods of regular training or recording sessions. During these periods, monkeys receive water or fruit juice as a feedback reward and reinforcer for performing the laboratory task, usually a visual discrimination task (Wegener et al., 2004; Grothe et al., 2012; Schledde et al., 2017; Fischer and Wegener, 2018). On non-working days (e.g., during the baseline period or at the weekend during laboratory periods), monkeys receive fruits and liquids in generous amounts (i.e., fully satisfying their physiological needs and allowing to compensate any possible deficit from laboratory periods). Fruits and fluids were available throughout the day, or were given once or twice for several hours, depending on the monkey’s individual protocol and the laboratory schedule of its group. Primate food pellets and seeds were available at all times. In the remainder of the paper, we refer to this schedule as “non-restricted.” Non-laboratory periods are defined as periods following at least 3 d of non-restricted food and liquid supply after a training or experimental block; body weight was taken regularly during baseline and non-laboratory periods and every working day during laboratory periods.

#### Other behavioral and physiological data

All monkeys were regularly assessed by an external veterinarian with long-term, laboratory and non-laboratory NHP experience, typically without preceding announcement of his visit. Monkeys were scored for 11 attributes of outer appearance and behavior. Note that on individual visits, one or more attributes were occasionally not scored because the monkey was engaged in a laboratory session, was not approaching close enough, or because of other reasons. Per assessed attribute, zero to three score points (SPs) were given (see Table 1 for scoring criteria). A total of four SP indicated the lower bound of overall “slightly disturbed” healthiness (i.e., mild severity). To assess individual monkeys, we calculated the ratio-to-mild as $rtm={\sum^{}}_{SP}/4$, with rtm=1 indicating the transition from “no complaint” to “mild severity.” Analysis of scores was performed (1) for individual monkeys, (2) for individual attributes, and (3) as function of time from date of implantation.

**Table 1:** Scoring attributes and criteria

Attribute	–	+	++	+++
Coat	Smooth, shiny	Rough, duff, protruding	Like + with gluing	Like ++ with loss of space
Skin	Smooth, physiological color	Dry, rough, scaly	Cracked, barked	Lesions
Orifices	Clean, dry	Light secretions	Soiling	Lesions
Nutritional status	Good	Moderate	Skinny, fat	Atrophy, obese
Mucosal membrane	Pale, pinkish red	Red	Washed out	Pale, cyanotic, yellow
Behavior at rest	Relaxed	Tense	Trembling	Nervous, anxious
Behavior in motion	Coordinated, fluent	Uncoordinated	Clear lameness and/or ataxia	Highly disturbed
Solitary behavior	Inconspicuous	Unphysiological repetitions/apathy	Stereotypical	Ethopathological
Social behavior	Cheerful, active	Passive, inhibited	Permanently threatened	Permanently aggressive/defensive
Behavior in presence of observer	Attentive	Indifferent	Angry, aggressive	Attempt to escape/massive attack
Behavior in presence of staff	Attentive	Indifferent	Angry, aggressive	Attempt to escape/massive attack

– equaled 0 SPs; +, ++, and +++ equaled 1, 2, and 3 SPs, respectively. A total of 4 SPs indicated slightly disturbed healthiness.

### Data analysis

All blood probes were sent to the same independent, commercial biomedical laboratory for cell counts, differential, and electrophoresis and were assessed by a veterinarian of the laboratory before dispatch. All blood values were transferred to Excel spread sheets on postal or E-mail arrival. Manually completed score sheets were also transferred to Excel spread sheets. Data analysis was performed by custom-written scripts in MATLAB 2020a (The MathWorks), using samples drawn for routine health checks but excluding samples drawn for veterinary purposes. Statistical testing was performed using functions from MATLAB’s Statistics and Machine Leaning Toolbox. False discovery rate (FDR) was calculated using the Benjamini and Yekutieli (2005) procedure (i.e., with no assumption on dependency structures), as provided in Groppe D (2021); fdr_bh (https://www.mathworks.com/matlabcentral/fileexchange/27418-fdr_bh), MATLAB Central File Exchange, retrieved May 21, 2021.

### Statistics

Significance is reported at *p *<* *0.05. With the exception of *rtm* over time, all other analyses were performed using non-parametric tests. If applicable, *post hoc* analysis was performed using Tukey’s HSD criterion. Ratio-to-mild *rtm* over time was tested by weighted regression, relying on *t* statistics, and weights were chosen by the number of data available per bin. For testing effects over the entirety of blood values, false positives were controlled by setting the FDR criterion to α = 0.1. Literature values for comparison were obtained from Stanley and Cramer (1968), Rosenblum and Coulston (1981), Kessler and Rawlins (1983), Bennett et al. (1992), Fernie et al. (1994), Buchl and Howard (1997), Hom et al. (1999), Smucny et al. (2001), Andrade et al. (2004), Chen et al. (2009), Didier et al. (2012), Koo et al. (2019), and Yu et al. (2019). If a given paper reported blood values separately for male and female animals, we only considered males. If a paper reported blood values separately for different age groups, we excluded values from outside the range of our own cohort (2–18 years). If applicable, means and SDs of literature values were weighted by the number of samples in each age group. Likewise, combined statistics across literature values were weighted by sample size *N* per study.

## Results

### Database

A total of 248 blood samples was drawn from 39 male rhesus monkeys. For each sample, the database included indexes on several criteria to characterize the reason for sampling, the presence of a cranial implant, the overall experimental conditions of the monkey delivering the sample (laboratory and non-laboratory periods), and anesthesia (see Materials and Methods). For each sample, the database also provided information about the time the sample was taken, the age of the monkey at the time of sampling, and, if applicable, the time of implantation and start of laboratory procedures of that individual. Combination of indexes and consideration of timing information allowed to differentiate suitable subsets of data for the different research questions to address. Indexes were used to identify the number of samples available for a given blood parameter or criterion as well as to identify the number of monkeys delivering these samples, and they underly all grouping of data in the remainder of the paper. An overview of the grouping will be provided in the following and the grouping will clearly be explained for each of the different analyses we perform.

**Figure 1.**
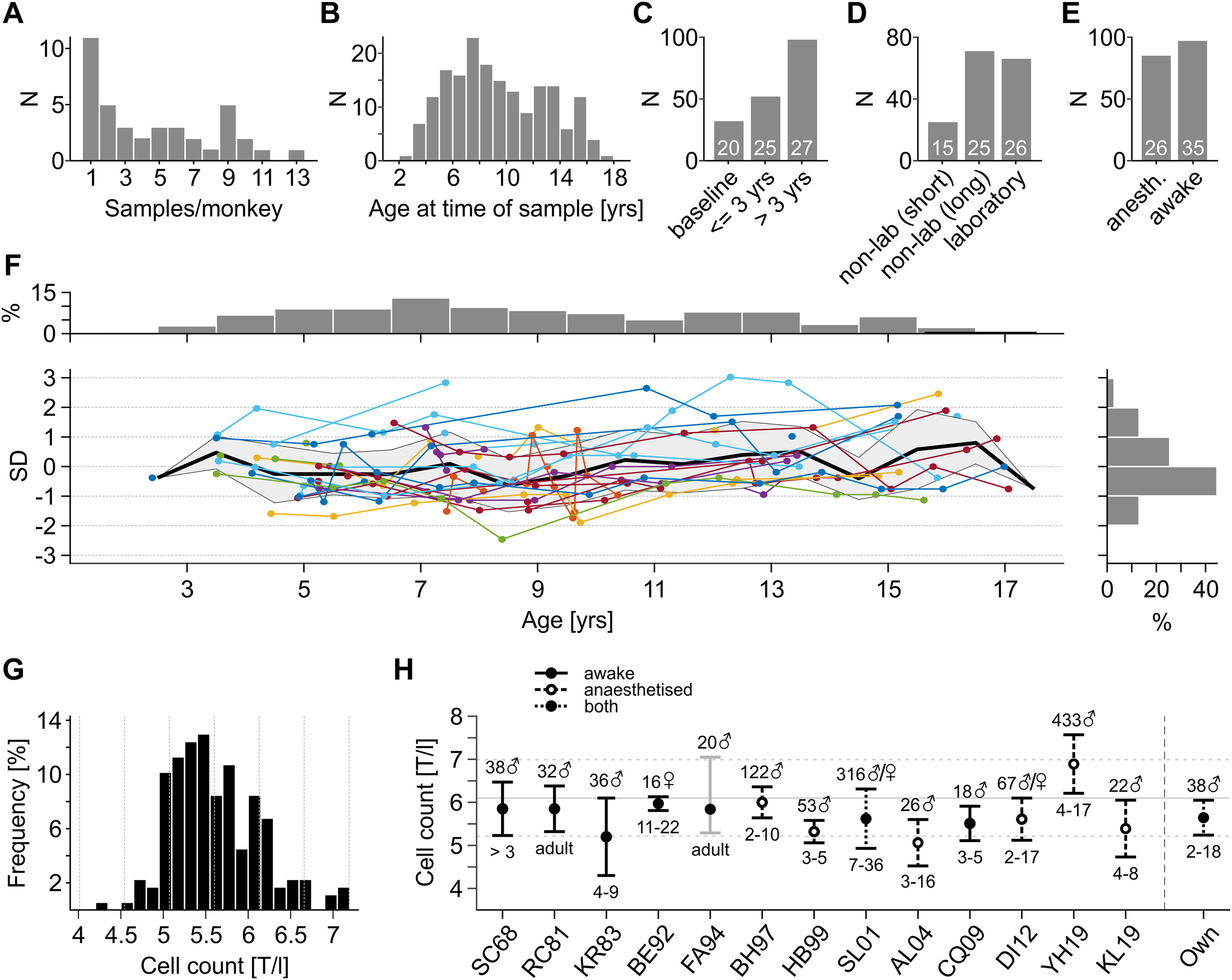
Database overview. ***A***, Number of monkeys providing between 1 and 13 individual samples. ***B***, Number of samples obtained at different age. ***C***, Number of samples from monkeys without cranial implant and before start of laboratory procedures (baseline), during first three years after implantation (≤3 years), and at later times (>3 years). ***D***, Number of samples taken during laboratory and non-laboratory periods. Non-laboratory periods are periods in-between blocks of laboratory work; “Non-lab short” indicates samples taken after 3–10 d of free access to fluid and food; “Non-lab long” indicates samples drawn after >10 d of free access. ***E***, Number of samples from anesthetized and awake monkeys. White numbers in ***C–E*** indicate the number of individual monkeys contributing to the data. ***F***, Statistical distribution of single red blood cell samples as function of monkey age, estimated over entire database. Colored lines correspond to samples from individual animals. Bold black line indicates mean over all monkeys per age. Gray-shaded area indicates 1 SD per age. Horizontal histogram indicates percentage of samples per age, vertical histogram indicates percentage of samples in terms of SD. ***G***, Distribution of red blood cell counts over all samples. T/l: 10^12^ cells/l. ***H***, Comparison of red blood cell counts to corresponding reports from the literature. Labeling of *x*-axis (SC68, …, KL19) refers to Stanley and Cramer (1968), Rosenblum and Coulston (1981), Kessler and Rawlins (1983), Bennett et al. (1992), Fernie et al. (1994), Buchl and Howard (1997), Hom et al. (1999), Smucny et al. (2001), Andrade et al. (2004), Chen et al. (2009), Didier et al. (2012), Koo et al. (2019), and Yu et al. (2019), in that order. “Own” refers to data from present study, as shown in ***G***. Signs on top of error bars indicate number and gender of animals, signs below error bars indicate age. Error bars indicate SD, with the exception of FA94, which represent data range (indicated by gray color of error bar).

Of all samples, *N *=* *66 (26.6%) were drawn because of veterinary purposes, e.g., as part of postoperative care. These samples were excluded from analysis. All other samples (*N *=* *182) were drawn for reasons of routine health check. Individual monkeys provided between 1 and 14 samples (Fig. 1*A*), and they were aged between 2 and 17 years at the time of sampling (Fig. 1*B*). A total of 32 samples from 20 animals were drawn before cranial implantation and start of laboratory procedures. These served as a baseline condition for various analyses; 52 samples from 25 individual monkeys were drawn during the first three years after implantation and start of laboratory procedures, and 98 samples from 27 monkeys were drawn at later times (Fig. 1*C*). Likewise, 66 samples from 26 monkeys were drawn during times the monkeys were currently engaged in laboratory procedures with limited access to fluid and fruits, 25 samples from 15 monkeys were drawn during times after or in-between blocks of laboratory work, after 3–10 d of free access to food and fluid, and 71 samples from 25 monkeys were drawn after longer non-laboratory periods (Fig. 1*D*). Finally, 86 samples from 26 animals were drawn during anesthesia, usually shortly after induction, and 96 samples from 35 monkeys were drawn from awake individuals (Fig. 1*E*). Anesthesia did not affect blood parameters in our database, as tested on all samples drawn before cranial implantation and start of laboratory (Kruskal–Wallis tests on 45 blood parameters, *p* values adjusted by FDR set to α = 10%, all *p *>* *0.11).

An illustrative example of the database is shown for red blood cells (Fig. 1*F*). The database delivered a total of *N *=* *177 routinely drawn red blood cell counts from 38 individual monkeys. ∼70.1%, 96.1%, and 99.4% of all samples were within 1, 2, and 3 SDs of the distribution, respectively, closely matching normal distribution, and 87.6% were taken from animals between 4 and 15 years of age. The mean red blood cell count was 5.6 ± 0.53 × 10^12^ units per liter (T/l; Fig. 1*G*). Comparison to the literature (see Materials and Methods) reveals good correspondence with the majority of published baseline values; yet it also reveals a good degree of variability of this rather basic hematologic parameter across studies, co-existing with differences in gender, age, sample size, state of anesthesia, and possibly other, hidden sources of variance (Fig. 1*H*). This introduces some uncertainty for the interpretation of blood values, particularly if they may be influenced by the specific husbandry and experimental conditions, and underlines the importance of a baseline reference drawn under basically identical overall conditions, to reduce variance across samples to some minimum. For the following analyses, therefore, possible long-term health effects because of the presence of cranial implants, controlled fluid supply, and potential stress arising from laboratory work were compared statistically to baseline values obtained from our own database, represented by samples from non-implanted animals before start of laboratory procedures (compare Fig. 1*C*, left bar). Literature values will be shown as complementary information.

### Effect of cranial implants

Because of various methodological reasons, a typical neuroscience experiment with awake behaving monkeys requires one or several permanent cranial implants (headpost, recording chamber, electrode connectors, or the like). Implants, materials, and surgical procedures are subject to continuous refinement (Adams et al., 2007, 2011; Galashan et al., 2011; McAndrew et al., 2012; Lanz et al., 2013; Johnston et al., 2016; Chen et al., 2017; Overton et al., 2017), yet the presence of an implant may increase the risk of bacterial or fungal infection at the site of implantation (Gardiner and Toth, 1999; Johnston et al., 2016). We were, therefore, asking whether blood samples drawn from animals with cranial implants would provide evidence for chronic inflammatory responses, both on the short and long term, as compared with a baseline condition, consisting of samples routinely drawn before implantation and start of laboratory procedures.

**Figure 2.**
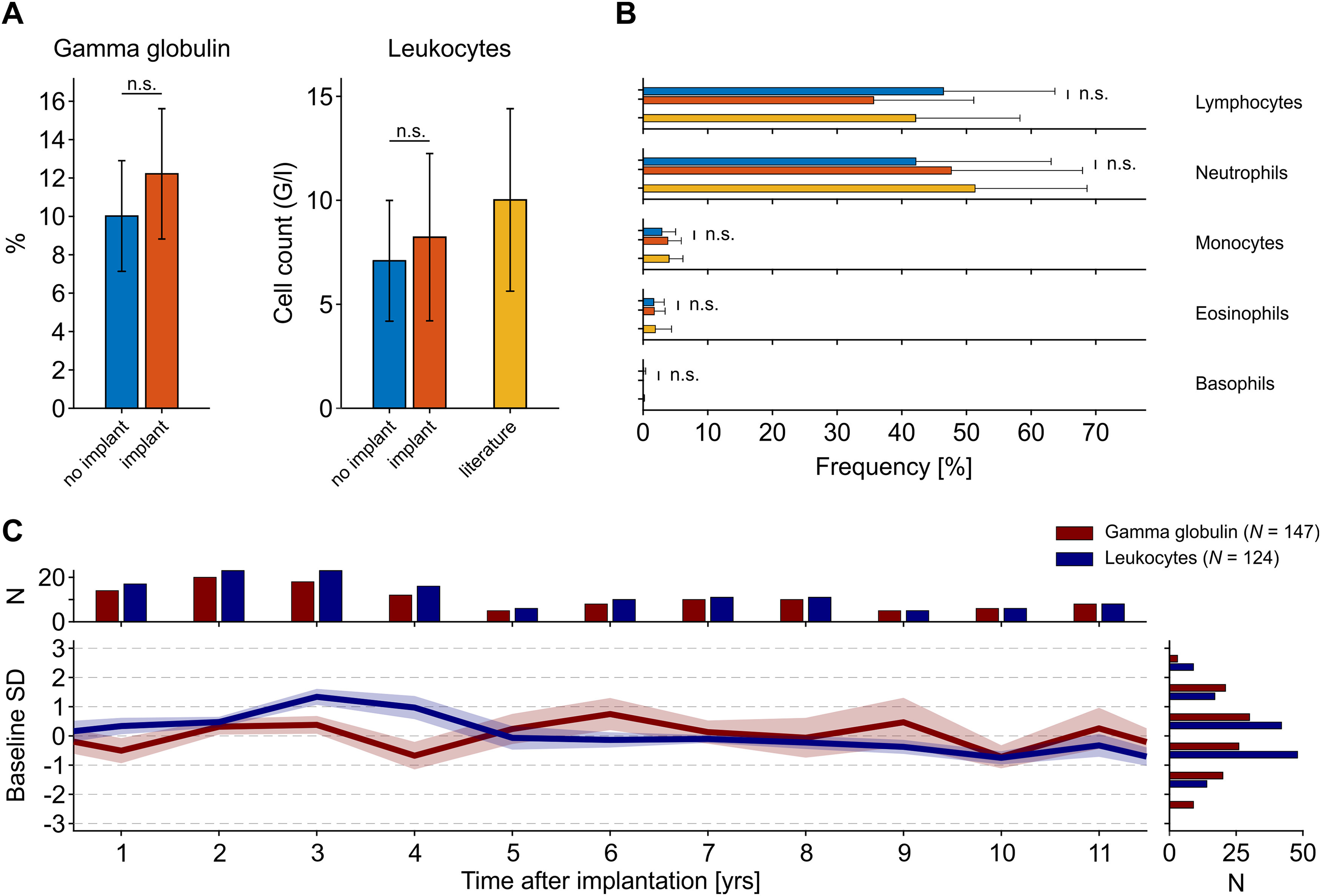
γ Globulin and leukocyte levels as markers of chronic inflammatory responses. ***A***, Mean γ globulin and leukocytes before and 6–18 months after implantation of a head cap, for leukocytes also compared with literature values (compare Fig. 1). Samples before and after implantation were taken from the same animals (*N *=* *11). Error bars for own data represent SD, error bars for literature values represent the range between the 2.5th and 97.5th percentile of published means. G/l: 10^9^ cells/L. ***B***, Percentage of the different leukocyte fractions before and after implantation, compared with literature. Error bars as in ***A***. ***C***, γ globulin and leukocyte levels for up to 11 years after implantation, expressed as mean deviation from overall mean and recalculated to the SD of baseline values before implantation (shown by blue bars in ***A***). Shading represents SEM; histograms indicate number of individual samples per year (top) and SD (right).

For 11 animals, the database provided both baseline samples and samples drawn between 6 and 18 months after implantation. These allowed for a paired analysis to ask whether, across monkeys, there is evidence for a consistent increase in γ globulin levels and leukocyte numbers, both being indicators of acute and chronic infections (Martin and Leibovich, 2005; Schroeder and Cavacini, 2010; Theil et al., 2021). We found that values after implantation were slightly higher than before implantation, yet neither for γ globulin nor leukocytes did this increase reach statistical significance (Wilcoxon signed-rank test, *p* = [0.175 0.153], *N* = [11 11] for γ globulins and leukocytes, respectively; Fig. 2*A*). The number of leukocytes was well in the range of published values (all acquired from non-implanted animals), both before and after implantation (Fig. 2*A*, right panel). The level of γ globulins could not be directly compared with the literature, because published values either do not consider globulins at all (Stanley and Cramer, 1968; Rosenblum and Coulston, 1981; Bennett et al., 1992; Andrade et al., 2004; Koo et al., 2019), consider total globulin (Kessler and Rawlins, 1983; Fernie et al., 1994; Buchl and Howard, 1997; Hom et al., 1999; Smucny et al., 2001; Chen et al., 2009; Didier et al., 2012; Yu et al., 2019), or report specific types of immunoglobulins (Yu et al., 2019), which consist mainly but not exclusively of IgG in the electrophoretic γ spectrum. Among leukocytes, we also tested the differential cell counts before and after implantation, but did not find any significant deviation (Wilcoxon signed-rank tests, all *p *>* *0.1, all *N *=* *11), all white blood cells were well in the range of published values (Fig. 2*B*).

If not evident on the short-term, chronic infections because of the presence of a cranial implant may build up on longer time scales, over the course of several years. Per year after implantation, the database provided between minimally five and up to 23 routinely drawn samples over a period of 11 years (total *N*: leukocytes: 124, γ globulin: 147). However, apart from small, year-wise fluctuations there was no obvious pattern to suggest any long-term changes in blood levels of leukocytes and γ globulin (Fig. 2*C*). Statistical analysis with the main factor “year after implantation” indicated no significant differences between γ globulin levels (Kruskal–Wallis test, χ^2^_(11,123)_ = 11.89, *p *= 0.37), and no value was different from baseline (all *p *> 0.084). For leukocytes, a significant main effect (χ^2^_(11,146)_ = 36.62, *p *<* *10^−3^) was found, which, however, was because of a smaller number of leukocytes at year 9 and 11 as compared with year 4 after implantation (*p *<* *0.024). No other pair-wise tests revealed significantly different levels of leukocytes (all *p *>* *0.067). Moreover, with the exception of the mean leukocyte value four years after implantation (*p = *0.007), no other value differed from baseline before implantation (all *p *>* *0.33).

### Effect of controlled fluid supply

In a typical systems neuroscience experiment, neuronal activity is recorded while monkeys engage in a cognitive task. Task performance is generally based on positive reinforcement protocols (Newsome and Stein-Aviles, 1999; Fischer and Wegener, 2018), using either food or fluid as the reinforcer. The choice of the reinforcer mainly depends on the experimental question, but fluid is the reinforcer of choice for the majority of studies. For the effectiveness of the reinforcer, monkeys are usually subjected to well-defined protocols of limited access during periods of laboratory work (lasting up to several weeks or months). Periods of laboratory work may be interrupted by non-laboratory periods (“holidays/vacations”; Prescott et al., 2010), during which monkeys are provided with fluid and food *ad libitum*.

Fluid protocols mainly differ regarding the amount of fluid provided outside the experimental session (Prescott et al., 2010). In the authors’ laboratory, monkeys are allowed to perform the behavioral task until they show no further interest in getting more reward, but they are not provided with additional fluids outside the laboratory. At weekends, during which animals usually do not work, they get individually tailored, generous amounts of fruits and fluids (see Materials and Methods). Besides behavioral observations and laboratory performance, body weight is measured all working days and adds to the information for specifying the individual protocol.

**Figure 3.**
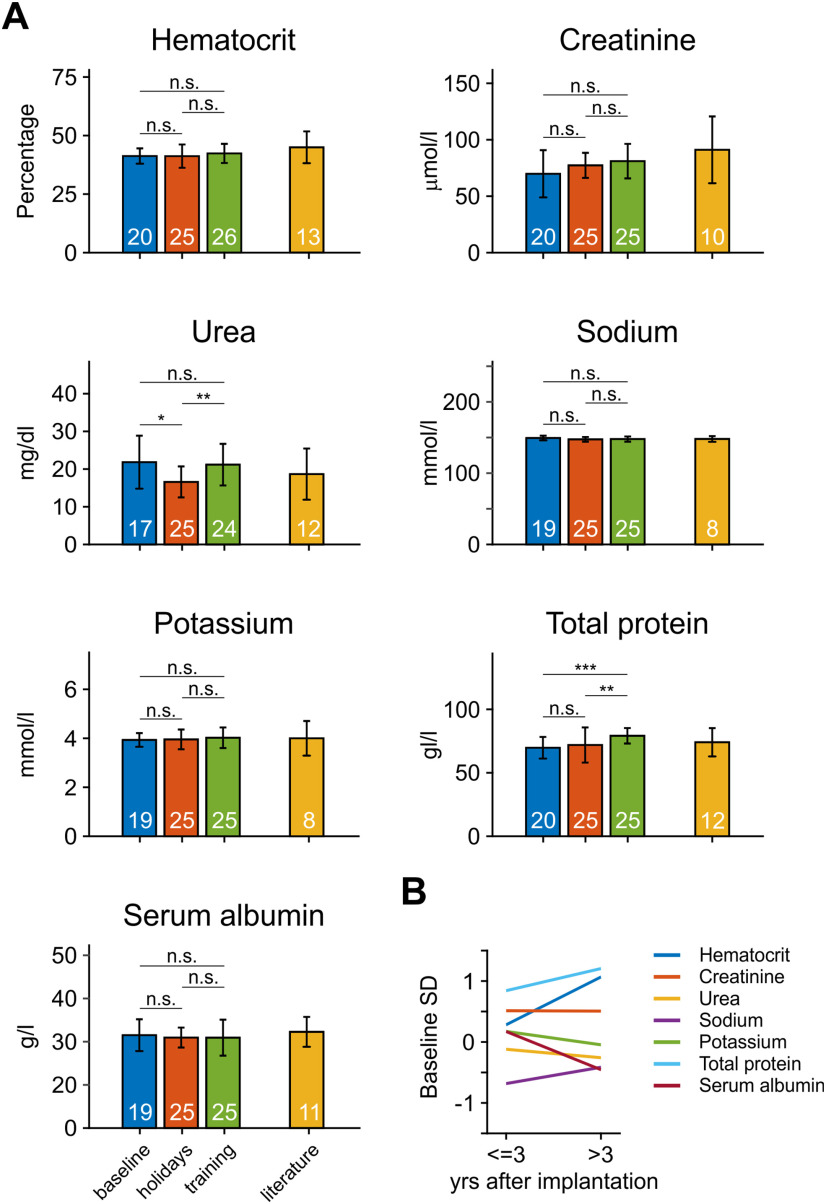
Blood parameters indicative of kidney function and osmolarity during periods of free and limited water supply. ***A***, Barplots show mean values of seven key parameters during baseline, holidays, and training, compared with literature values (right-most bars). n.s., not significant; *p <* *0.05, **p <* *0.01, ***p <* *0.001. Error bars indicate ±1 SD. Numbers in bars provide the *N* of monkeys contributing to the data or the *N* of research papers contributing to literature values. ***B***, Parameters of the training group, subdivided into early (≤3 years after implantation) and late (>3 years after implantation) samples, expressed as fractions of baseline SD.

To test whether monkeys regulate their fluid balance appropriately to maintain their health, we analyzed the hematocrit, creatinine, urea, sodium, potassium, and total protein as indicators of kidney function, and serum albumin as indicator of colloid osmotic pressure (Hall, 2015). All parameters were categorized into three groups: (1) “baseline,” consisting of samples routinely taken during periods of non-restricted water supply, drawn before any laboratory training; (2) “holidays,” consisting of samples routinely taken during non-laboratory periods after at least 10 d of non-restricted fluid supply; and (3) “laboratory,” consisting of samples routinely taken during laboratory periods with limited fluid supply. The database provided between 24 and 68 samples per parameter and group (Fig. 3*A*). To avoid a bias from animals with numerous samples, samples of each group were averaged per monkey, resulting in *N *=* *17–26 values per parameter and group; *p* values of Kruskal–Wallis tests over the three groups of samples were adjusted to conform to a FDR of 10%. Statistical analysis indicated no effect for hematocrit, creatinine, sodium, potassium, and serum albumin (all *p *>* *0.15), but a main effect for urea (*p *=* *0.015) and total protein (*p *<* *10^−3^). Pair-wise tests showed that the urea level of the holidays group was significantly smaller than those of the baseline (*p *=* *0.004) and the laboratory (*p *=* *0.014) group, while the two latter were not different from each other (*p *=* *0.77). For total protein, there was no difference between baseline and holidays (*p *=* *0.27), but the laboratory group showed a significantly larger level than the other groups (*p *<* *0.009). For all groups and parameters, values were clearly in the physiological range, as obtained from reference data of non-deprived animals (Stanley and Cramer, 1968; Rosenblum and Coulston, 1981; Bennett et al., 1992; Buchl and Howard, 1997; Hom et al., 1999; Smucny et al., 2001; Andrade et al., 2004; Chen et al., 2009; Didier et al., 2012; Koo et al., 2019; Yu et al., 2019).

In addition to comparing between baseline, holiday, and laboratory periods, we further tested whether parameters indicative of hydration may slowly deteriorate over the course of years and subdivided the laboratory group into two temporal groups. The first group consisted of samples drawn during the first three years after implantation and start of laboratory procedures (*N *=* *18 for all parameters) and the second group consisted of all samples drawn thereafter (*N *=* *14–16). To directly relate these values to the baseline condition before any laboratory work (*N *=* *17–20), we subtracted the mean value of the baseline group from the mean values of the two laboratory groups and expressed the difference in terms of baseline SD (Fig. 3*B*). For all tested parameters, mean values stayed within ±1 SD, independent of the time they were drawn. With the exception of total protein (*Z *=* *2.07, *p *=* *0.038, *N* = [18 15]), no other parameter showed a significant difference between samples drawn during the first three years and samples drawn during later years (Wilcoxon rank-sum tests, all *Z *<* *1.9, all *p *>* *0.1).

### Effect of laboratory procedures

The training of cognitive tasks and the conduction of neurophysiological recordings typically involves temporarily removing an animal from its social group, usage of a primate chair, transport from husbandry to laboratory, restraining by head-fixation, and preparation of recording equipment, as, e.g., mounting a microdrive and lowering down the electrode. These laboratory procedures, together with controlled water or fluid supply, have been discussed to potentially induce distress to the animal in European and United States regulatory and welfare documents (European Parliament, 2010; National Research Council, 2011). However, because the presentation of a complex cognitive task to a distressed animal would have considerable drawbacks when conducting neurophysiological research, an important goal for the refinement of laboratory procedures has been the quantitative assessment of characteristic stress markers. Among those, the level of salivary or plasma cortisol, the primary glucocorticoid in humans and NHPs, has been investigated with respect to social separation, social rank, movement restraint, and fluid deprivation in macaque monkeys (Sapolsky and Mott, 1987; Clarke et al., 1988; Higley et al., 1992; Pfefferle et al., 2018). An alternative to measuring cortisol is to determine the neutrophil/lymphocyte (N/L) ratio in blood samples. Because the biosynthesis of both neutrophils and lymphocytes is modulated by stress hormones (Deitch and Bridges, 1987), the N/L ratio is suggested as an indirect measure of plasma stress hormone levels and was shown to positively correlate with cortisol (Kim et al., 2005; Lee et al., 2013).

**Figure 4.**
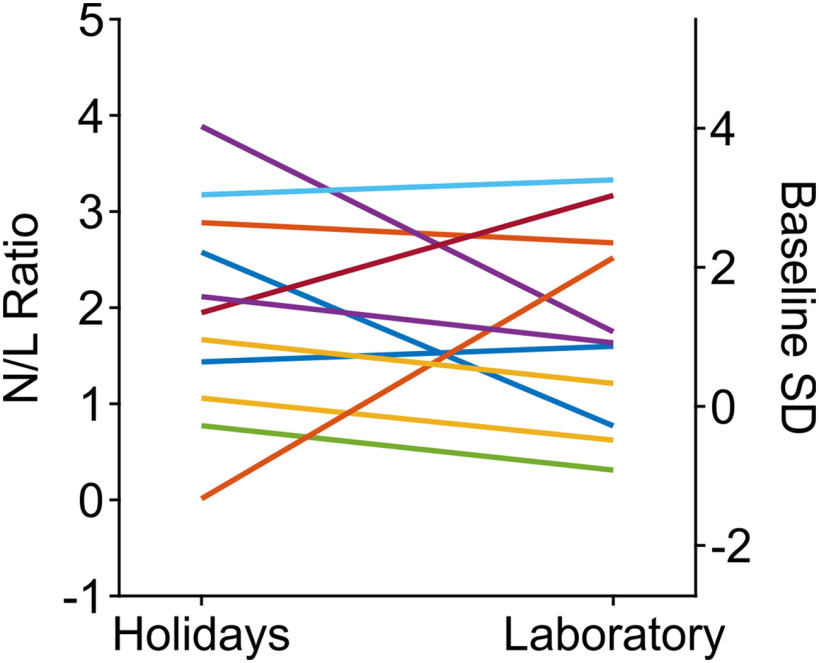
N/L ratio (left *y*-axis) during periods of holidays and laboratory periods of *N *=* *11 monkeys (colored lines), expressed as fractions of baseline SD (right *y*-axis). Per monkey, baseline, holiday, and laboratory periods are represented by means whenever more than one sample was available.

We used the N/L ratio to ask for evidence on increased levels of distress during laboratory periods. Eleven monkeys delivered blood samples during baseline periods before implanting a cranial cap and start of laboratory work, during laboratory periods, and during holiday periods following at least 10 non-laboratory days. Note that monkeys also receive care during holidays, such that they may be taken to the lab for a short time every second or third day. Statistical analysis of this data revealed no significant difference but a trend for differences between the three groups (Kruskal–Wallis test, χ^2^_(2,30)_ = 5.45, *p *=* *0.066). *Post hoc* analysis showed a tendency for slightly higher N/L ratios during holiday (*p *=* *0.07) but not laboratory periods (*p *=* *0.18) compared with baseline. A direct comparison between holiday and laboratory periods showed that the N/L ratio stayed the same for some individuals, for some others it slightly increased during laboratory periods, and for still some others it slightly decreased (Fig. 4). On the group level, there was no indication for a difference between the two groups (*p *=* *0.9).

### General health assessment

Table 2 provides an overview about the entirety of blood values, sorted into three groups: blood values of animals before implantation and start of laboratory procedures (group 1), blood values of animals during the first three years after implantation and start of laboratory procedures (group 2), and blood values of animals at times later than three years after implantation and start of laboratory procedures (group 3). Multiple samples of individual animals were averaged per group, such that each monkey was weighted equally. Per blood value, samples were compared by Kruskal–Wallis tests and, if applicable, paired *post hoc* tests; *p* values of Kruskal–Wallis tests were adjusted to conform to an FDR of 10%.

**Table 2:** Full list of blood parameters over time

Param	Unit	Group 1 (baseline)			Group 2 (implant ≤3 years)			Group 3 (implant >3 years)			KW	Paired tests (groups)		
		Mean	SD	*N*	Mean	SD	*N*	Mean	SD	*N*		1–2	1–3	2–3
Hemogram														
Leukocytes	G/l	8.38	2.6	20	9.62	2.95	25	10.19	3.66	27	n.s.	-	-	-
Erythrocytes	T/l	5.56	0.44	19	5.54	0.41	25	5.73	0.44	27	n.s.	-	-	-
Hemoglobin	g/l	131.71	7.49	20	131.46	9.8	25	135.55	12	27	n.s.	-	-	-
Hematocrit	%	40.86	2.81	20	41.85	2.82	25	42.81	3.94	27	n.s.	-	-	-
MCV	fl	73.95	4.29	19	75.64	4.3	25	74.89	4.81	27	n.s.	-	-	-
MCH	pg	23.96	1.46	19	24.1	1.21	25	23.75	1.61	27	n.s.	-	-	-
MCHC	g/dl	32.45	1.25	19	31.83	1.04	25	31.74	0.99	27	n.s.	-	-	-
Thrombocytes	G/l	369.76	76.29	19	354.43	77.13	25	354.17	101.16	27	n.s.	-	-	-
Differential														
Basophils	%	0.11	0.31	20	0	0	25	0.07	0.21	27	n.s.	-	-	-
Eosinophils	%	1.38	1.43	20	1.65	1.34	25	1.45	0.99	27	n.s.	-	-	-
Lymphocytes	%	45.53	18.33	20	39.61	13.69	25	34.02	14.24	27	n.s.	-	-	-
Monocytes	%	3.41	2.6	20	3.6	1.77	25	4.36	2.26	27	n.s.	-	-	-
Neutrophils	%	45.82	21.4	20	54.63	13.56	25	57.59	18.8	27	n.s.	-	-	-
T3	μg/l	1.82	0.43	18	2.15	0.63	24	1.82	0.59	25	n.s.	-	-	-
D3	nmol/l	171.01	48.96	16	156	75.16	23	147.42	44.23	23	n.s.	-	-	-
Electrophoresis														
Albumin	g/l	44.7	2.86	5	44.23	2.2	5	44.13	4.32	17	n.s.	-	-	-
α1-globulin	g/l	2.67	0.41	6	2.47	0.32	6	2.66	0.64	17	n.s.	-	-	-
α2-globulin	g/l	8	2	6	8.94	2.46	6	9.17	1.73	17	n.s.	-	-	-
β-globulin	g/l	7.75	2.52	6	8.62	1.89	6	10.13	1.82	17	n.s.	-	-	-
γ-globulin	g/l	7.58	3.64	6	6.81	3.23	6	10.04	2.45	17	n.s.	-	-	-
Chloride	mmol/l	108.32	1.98	17	106.6	2.9	24	107.49	2.64	26	n.s.	-	-	-
Iron	μmol/l	25.21	6.57	19	24.04	5.75	25	23.96	12.02	27	n.s.	-	-	-
Geriatric profile														
T4	μg/dl	4.44	1.25	18	4.43	1.31	24	4.87	1.28	26	n.s.	-	-	-
Kidney														
Urea	mg/dl	21.97	6.89	18	19.67	5.56	25	18.03	5.53	26	n.s.	-	-	-
Creatinine	μmol/l	69.4	17.81	20	77.69	12.65	25	82.46	14.56	27	n.s.	-	-	-
Total protein	g/l	69.76	8.6	20	75.53	6.36	25	75.25	14.74	27	n.s.	-	-	-
Sodium	mmol/l	149.07	2.92	19	147.16	3.59	25	148.71	3.31	27	n.s.	-	-	-
Potassium	mmol/l	3.91	0.27	19	3.98	0.35	25	3.96	0.45	27	n.s.	-	-	-
Phosphates	mmol/l	1.56	0.35	19	1.44	0.33	25	1.32	0.29	27	n.s.	-	-	-
Liver														
Bilirubin	μmol/l	2.69	0.78	20	2.9	1.08	25	3.2	1.23	27	n.s.	-	-	-
ALT	U/l	84.27	88.26	19	63.75	41.24	25	80.67	104.23	27	n.s.	-	-	-
AP	U/l	420.52	204.04	19	339.6	208.56	25	216.23	81.65	27	**	n.s.	***	**
γ-GT	U/l	75.47	27.08	19	68.39	22.04	25	60.16	23.5	27	n.s.	-	-	-
AST	U/l	43.92	17.04	19	38.15	24.1	25	31.17	14.2	27	n.s.	-	-	-
GLDH	U/l	38.58	46.8	19	20.75	13.24	25	36.7	53.69	27	n.s.	-	-	-
Serum albumin	g/l	43.06	7.37	19	44.07	4.29	25	40.22	8.02	27	n.s.	-	-	-
Pancreas														
Glucose	mmol/l	4.82	1.34	20	4.38	1.06	25	4.66	1.34	27	n.s.	-	-	-
α-Amylase	U/l	468.11	105.71	19	495.52	119.96	25	461.74	144.65	27	n.s.	-	-	-
Lipase	U/l	22.34	6.72	18	18.55	3.47	25	20.18	7.36	27	n.s.	-	-	-
Cholesterol	mmol/l	3.57	1.06	20	3.26	0.52	25	3.54	0.77	27	n.s.	-	-	-
Muscle														
CK	U/l	174.02	74.57	19	200.53	208.21	25	167.36	119.89	27	n.s.	-	-	-
LDH	U/l	402.31	146.41	19	266.37	92.61	25	260.89	79.26	27	*	***	***	n.s.
Calcium	mmol/l	2.41	0.11	19	2.43	0.14	25	2.47	0.14	27	n.s.	-	-	-
Magnesium	mmol/l	0.74	0.07	19	0.72	0.09	25	0.78	0.08	27	n.s.	-	-	-
Triglycerides	mmol/l	0.65	1.18	19	0.63	0.38	25	1.03	0.49	27	***	*	***	**

Mean values, SD, and number of samples *N* are given separately for time periods before implantation and start of laboratory procedures (baseline, group 1), during the first three years after implantation and start of laboratory procedures (group 2), and later times (group 3). Column KW provides results of Kruskal–Wallis tests; *p* values were adjusted to conform to a FDR of 10%. Column paired tests indicates results of *post hoc* tests when Kruskal–Wallis tests rejected the null hypothesis of equal means. n.s., not significant; *p <* *0.05, **p <* *0.01, ***p <* *0.001. U/l, units/l; G/L, 10^9^ U/l; T/l, 10^12^ U/l.

Among 45 blood parameters, three parameters were found to be significantly different. Alkaline phosphatase (AP) of both group 1 and group 2 was higher than of group 3 (*p *<* *0.01), lactate dehydrogenase (LDH) of group 1 was higher than of both group 2 and group 3 (*p *<* *0.001), and triglycerides were different between all groups (*p *<* *0.044), with group 3 having the highest mean value. AP represents one of seven available liver values, LDH and triglycerides represent two of five muscle values. None of the parameters from the hemogram, differential, and electrophoretic analysis and none of the parameters for kidney and pancreas function was found to deviate significantly between the three groups.

### Outer appearance and behavior

The hematologic analysis of cumulative effects on health and well-being was supplemented by an analysis of the individual score sheets of the monkeys, prepared by an external veterinarian (M.B.). Only monkeys that already participated in laboratory procedures were considered (*N *=* *34), to avoid any bias caused by animals that never had participated in laboratory procedures. Individual monkeys were scored between 21 and 122 times, and the overall database provided a total of *N *=* *2375 score sheets. Each monkey was assessed for five attributes of outer appearance and six attributes of behavior and was scored between 0 and 3 points per attribute, resulting in a total of 33 points possibly given (Table 1). A sum of four points over all 11 attributes marks the lower bound of an overall mildly disturbed healthiness.

**Figure 5.**
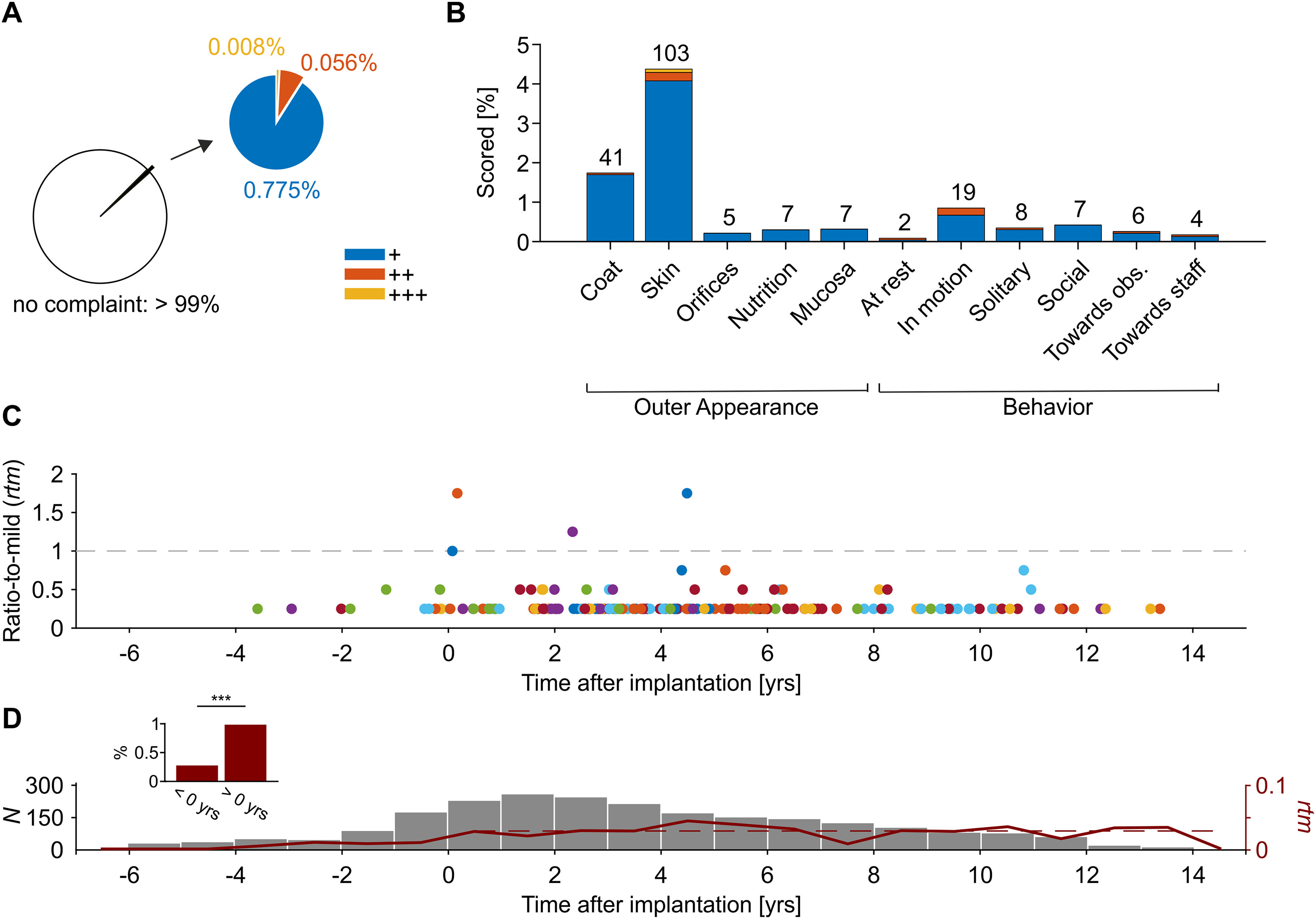
Summary of score sheets. ***A***, Score sheets with at least one scored attribute as ratio of all score sheets (lower left) and as ratio of score sheets with one (+) to three (+++) SPs per attribute (upper right). ***B***, Ratio of scores for individual attributes. Note that total *N* was different among attributes (because not always could a monkey been scored regarding all attributes). ***C***, Ratio-to-mild *rtm* for all score sheets with a sum >0 points (*N *=* *209), aligned to date of implantation. Dashed line indicates transition from “no complaint on healthiness” to “mildly disturbed healthiness” at *rtm *=* *1. Colors indicate different monkeys. ***D***, Total number of score sheets per year, before and after implantation. Red line indicates mean *rtm* per year (right *y*-axis). Dashed red line indicates weighted linear regression over mean *rtm* after implantation and start of laboratory work (slope <10^−5^, *p *=* *0.997). Inset shows sheets with score sum >0 as ratio of total score sheets before and after implantation and start of laboratory procedures; ***p < 0.001.

In total, 24,913 attributes were scored; 24,704 (99.16%) of these were without complaint (–), 193 were given one point (+), 14 were given two points (++), and two were given three points (+++; Fig. 5*A*). Each of the 11 attributes was scored at least twice in the total dataset, and none was scored >103 times (Fig. 5*B*). To investigate whether the frequency of scores, or their overall sum, increased as a function of time after start of laboratory procedures, we plotted the ratio-to-mild (*rtm*) of all score sheets, aligned to the year of implantation as temporal marker for the start of laboratory work (Fig. 5*C*). The ratio-to-mild equals rtm=1 at the transition from “no complaint” to “mildly disturbed healthiness” and rtm=2.5 at the transition to “moderately disturbed healthiness.” The results show that all but four score sheets were below the lower *rtm*-bound, indicating “no complaint” on overall healthiness. The remaining four score sheets came from three different monkeys and were indicating “mildly disturbed healthiness” (maximal *rtm *=* *1.8) at the time of scoring. No monkey was ever scored as “moderately disturbed.”

Regarding the question of cumulative and long-term effects, we found that monkeys were more frequently scored after start of laboratory work (χ^2^ test, χ^2^ = 23.43, *p *<* *10^−5^), but the effect was very small and conformed to a total of 13 scored attributes out of 4835 (0.27%) before implantation and start of laboratory work, and 196 out of 20,078 (0.98%) thereafter, and an increase from mean *rtm *=* *0.007 to mean *rtm *=* *0.028, respectively. After implantation, there was no increase in *rtm* over time (linear regression, weighted by number of score sheets per year, *p *=* *0.997; Fig. 5*D*).

## Discussion

The present paper investigates the combined, short-term and long-term influence of typical NHP neuroscientific procedures on hematologic values and general health conditions. The results provide no consistent statistical indication that the presence of a cranial implant, the application of controlled water supply, and exposure to laboratory procedures during training and experimental sessions cause a systematic deviation from baseline and literature levels, and they do not provide evidence of cumulative and long-term effects, or a combination of both. The analysis considered blood sample data taken during a life-span of up to 11 years following cranial implantation and start of laboratory procedures and was complemented by supplementary physiological and behavioral data providing identical conclusions. Albeit absence of statistical evidence is not principally excluding the existence of long-term, cumulative effects, the results of the study are in line with a number of recent investigations on potential health effects of NHP experimental procedures, and they underline the effectiveness of the many efforts being taken to optimize them.

### Impact of cranial implants

Cranial implants for mounting headpost and recording chambers were introduced in the early days of neurophysiological studies in the awake, behaving monkey (Evarts, 1968; Wurtz, 1968) and represent, for various reasons, a key technique for the advance of modern neuroscience. For long time, cranial implants typically consisted of a cap of dental acrylic with embedded headpost and recording chamber, anchored by medical screws. Although a generally well compatible technique, a drawback is that granulation tissue sometimes tends to grow at the skin margins of the cap, slowing down postsurgical healing and facilitating the risk of infections. In the worst case, these may become chronic. Refinements of the technique aimed at reducing the risk of infections: several laboratories introduced acrylic-free implants, formed and fabricated based on the individual monkey’s cranial anatomy and made from biocompatible materials to support complete osseous-integration (Adams et al., 2007, 2011; McAndrew et al., 2012; Lanz et al., 2013; Johnston et al., 2016; Chen et al., 2017; Overton et al., 2017). Additional refinements were introduced by imaging technologies like magnetic-resonance and computer tomography for, e.g., presurgical planning (Basso et al., 2021), as well as by techniques for postsurgical care as, e.g., the introduction of adaptable implant covers to protect the sutured skin (Perry et al., 2021).

We here add a modification of the surgical procedure for implanting acrylic caps to the list of refinements for cranial implants (see Materials and Methods). We have gained good experience with this technique and obtained cranial implants that show little signs of irritated tissue at the cap margins and require only minimal care over many years. For two key markers of chronic infections, leukocytes and γ globulin, our data do not reveal increased levels after implantation of the cranial cap as compared with before. We investigated a postimplantation time span of 11 years and found that leukocyte and γ globulin levels always stayed within 2 SDs, mostly within 1 SD, of the baseline distribution obtained before implantation. Because small infections at the margins of the cap can never be fully excluded, neither with our implants nor (presumably) with acrylic-free implants, this is suggesting that inflammatory responses, as they may arise occasionally, stay local and do not manifest systemically.

This conclusion is, however, different from the conclusion of two other, recent studies on long-term effects of cranial implants. Frydman et al. (2017) reported lower albumin and calcium concentrations in blood samples of macaques with implants as compared with those without, and also reported higher concentrations of globulin and creatinine. Based on additional investigations, the authors related these effects to the leakage of metal ions from titanium alloy cortical screws used to anchor the cranial implant and/or the recording chamber. Albumin, calcium, globulin and creatinine were found to either correlate with the number of implanted screws or the duration of implantation, or with both. Theil et al. (2021) applied statistical modeling techniques on blood samples of 57 macaques and found that implants were a significant predictor of changes in the number of neutrophils, lymphocytes, and red blood cells, and in the concentrations of hemoglobin, AP, creatinine, calcium, phosphorus, total protein, albumin, and globulin. These results are clearly different from the results of the present study: we found a significant difference between implanted and nonimplanted animals for only three of 45 parameters (AP, LDH, and total protein). We applied a rather liberal FDR of 10%, whereas Theil et al. (2021) used a rather conservative modification of the FDR to account for multiple comparisons. Although our approach increases the expected number of positive test results, it was, nevertheless, much smaller than in Theil et al. (2021). A reason for the discrepancy between these studies and ours might be given by the reduced risk of infection because of the refinement of the surgical procedure, as pointed out earlier. Another reason, however, might be given by more general factors, such as rearing, social experience, housing, enrichment, and stability of social relations; all of which are suggested to have a significant, long-term influence on physiological and immunologic parameters (Capitanio, 1998; Schapiro et al., 2000; Buchanan-Smith, 2010; Hannibal et al., 2017). The paper of Frydman et al. (2017) does not provide information about housing and social conditions, and the paper of Theil et al. (2021) only mentions that monkeys were kept singly or paired in cages. Monkeys in the authors’ institute, however, are housed in established social groups, they have large enclosures equipped with many different objects for climbing, exploring, playing, and have daily access to non-climate controlled outdoor areas, basically fulfilling all requirements of good primate housing as identified by the NC3R Joint Working Group on Refinement (Jennings et al., 2009). We believe that state-of-the-art keeping and housing and versatile social conditions will generally support immune competence and physiological healthiness, and eventually allow monkeys to better cope with the specific experimental situations than under more deprived conditions.

### Impact of controlled fluid supply

Fluid control protocols serve to provide an effective reinforcer during training and performance of cognitive tasks and as a general motivational incentive. Fluid control protocols in NHP laboratories span a relatively wide range, at least partly depending on local legislative regulations (Prescott et al., 2010): at one end, protocols must provide daily *ad libitum* access to water for a limited period of time on working days and free access to water on non-working days. At the other, fluid access might be limited to laboratory performance on working days and to individually tailored amounts on non-working days. A recent investigation of two different fluid protocols (a moderate and a rather strict one) with a control condition of *ad libitum* supply indicated only minor differences after 16 weeks of application (Gray et al., 2019). Urine levels of urea and creatinine, and urine specific gravity did not differ between the two fluid control protocols, but the urine was more concentrated than during periods of free access. Blood levels of urea, sodium, and creatinine, and the hematocrit, however, were the same as during free access. Ethograms derived from videotaped material did not provide indication of increased anxiety or other consistent behavioral observations to suggest a decrease in well-being during limited fluid supply, in line with another study (Hage et al., 2014). Both studies concluded that fluid control has less of an impact than often proposed.

In the authors’ laboratory, the standard approach is to provide fluid as reward for task performance on working days and individually tailored amounts of fruits and water on the weekend, starting Friday afternoon. Schedules for individual monkeys may include *ad libitum* supply of water. Schedules are based on task performance on Mondays and body weight: because monkeys are known to work longer and consume more water when they are in low hydration (Yamada et al., 2010; Minamimoto et al., 2012; Gray et al., 2016) and because body weight correlates with hydration (Yamada et al., 2010), below-average uptake of water on Mondays and steady average body weight/week on the long-term supports the conclusion of a well-balanced hydration of the respective individual. The blood values obtained with this protocol support this conclusion. We found that hematocrit, creatinine, urea, sodium, and potassium were not different between baseline and laboratory periods, and we found that all values were well in the range of physiological reference values from non-deprived animals. Urea was slightly lower during holidays, indicating well hydration, and holiday levels of the other parameters were just as during baseline and training. Only for total protein, we found a significant difference between laboratory and baseline/holidays, but absence of a difference between early and late periods following implantation and start of laboratory procedures (compare Table 2). Total protein provides information about the blood levels of albumins and globulins. Abnormal levels could point to dehydration or chronic infection, or a malfunction of kidney or liver. With regard to total proteins, dehydration would be signaled by albumins, which are responsible for 80% of the colloid osmotic pressure. Yet, albumins neither differed between baseline, holidays, and laboratory periods (Fig. 3) nor with regard to time after implantation (Table 2). Chronic infection, in turn, would be signaled by globulins. In neither spectrum did we find an increase of globulins over time (Table 2), especially not for γ globulins (Fig. 2). All typical kidney parameters were in the normal range, as were all values for liver, with the exception of AP, which was lower at later times, yet fully in the physiological range (Table 2). Thus, the increase in total protein during training periods is a stand-alone finding that does not coincide with other parameters to indicate dehydration, chronic infection, or a malfunction of liver or kidneys.

The finding that monkeys had adapted well to the fluid schedule is not too surprising given their feeding ecology in natural habitats. Macaques, as many other NHPs, live in arboreal and terrestrial regions, where periods with considerable rainfall may be followed by dry seasons with no water in rivers and ponds, and monthly rainfall of only a few millimeters (Neville, 1968; Lindburg, 1977; Makwana, 1978; Takenaka, 1986). During these times, they are limited to consume water from tree holes and to lick dew and rainwater in the early morning (Sharma et al., 2016). Note that this may coincide with temporary increases of plasma proteins, sodium, and creatinine (Takenaka, 1986). Even in rain season, macaques often refrain from visiting water holes during daytime, or for several days, because of the presence of predators (Lindburg, 1977; Amoroso et al., 2020). Thus, because of the periodic availability of water and for reasons of predator avoidance, macaques, as many other NHP species, have evolved to balance their water intake and maintain their health under conditions of periodical and limited water supply. Laboratory fluid protocols, therefore, are not expected to constitute an unknown physiological challenge, as shown by the absence of detectable changes regarding kidney function, water balance, or general health in our data. Given that (1) task requirements in the laboratory are accessible for the monkey to obtain its desired amount of liquid, (2) behavior and body weight is continuously monitored to allow identification of low hydration, (3) possible deficiencies may be compensated on non-training days, and (4) handling and overall laboratory conditions prevent anxiety and stress, there is also not much reason to assume a priori an impairment in psychological well-being during periods of controlled fluid supply, in line with the behavioral scores we reported here, and the ethograms of previous studies (Hage et al., 2014; Gray et al., 2016).

### Impact of laboratory procedures on well-being

Psychological well-being of laboratory monkeys depends on factors ranging from general housing conditions over enrichment to actual laboratory procedures, including man-monkey interactions at all levels (National Research Council, 1998; Coleman and Novak, 2017). A bunch of literature is available regarding experiences and results for keeping of monkeys in pairs or social groups (Novak and Suomi, 1991; DiVincenti and Wyatt, 2011; Truelove et al., 2017), providing intense sensory, social, and cognitive stimulation (Platt and Novak, 1997; Fagot and Paleressompoulle, 2009; Berger et al., 2018), and using positive reinforcement techniques for voluntary participation in husbandry, veterinary, and research procedures (Prescott and Buchanan-Smith, 2003; Scott et al., 2003; Kemp et al., 2017).

For conducting laboratory procedures, a central goal is to prevent anxiety and minimize distress, for the merit of both the well-being of the animal and the validity of the research data. Distress may occur, however, during initial training of animals: in a recent survey among international NHP laboratories (both governmental/academical and contract research/pharmaceutical sector), about half of all respondents considered some signs of distress during initial training for chair restraint, while only a few laboratories did so for well-trained animals (McMillan et al., 2017). With regard to physiological stress markers, serum cortisol levels during initial chair restraint training were found to be elevated, but were also found to return to baseline after three weeks of training (Lee et al., 2013). Salivary cortisol levels were found to be increased in animals under controlled fluid supply and in implanted animals during care of skin margins, but did not show cumulative effects when caring was applied to animals with controlled fluid access (Pfefferle et al., 2018). Also, the total increase in salivary cortisol was only a fraction of normal daily fluctuations and was thus interpreted as indicating a mild stress response.

We here add data on the long-term effect of laboratory procedures in rhesus macaques. All of the monkeys were implanted and were regularly taken away from their enclosure and their social group to receive care for skin margins and/or perform training or experimental sessions, they were under controlled fluid supply during laboratory periods, and they were subjected to procedures related to data acquisition (e.g., opening the recording chamber, mounting electrode drives, lowering down electrodes, etc.). On the group level, we found that the N/L ratio did not differ between holidays and laboratory periods, and, importantly, also did not differ in comparison to baseline. On the level of individuals, some monkeys showed a slightly increased N/L ratio and some others showed a slightly decreased one, but most had about the same ratio between holidays and laboratory periods. All values were in the physiological range described for humans (Forget et al., 2017), with the exception of two values of the holiday period, one being slightly too high and the other one slightly too low.

In macaque monkeys, the N/L ratio was recently shown to closely correlate with serum cortisol levels and was suggested as an indirect psychophysiological indicator for distress (Kim et al., 2005; Lee et al., 2013), because of the modulation of lymphocyte and neutrophil activity by stress hormones (Deitch and Bridges, 1987). Because it is a response to stress hormones, the N/L ratio has a slower time course. Continuous injections of cortisol were shown to significantly increase the level of circulating neutrophils after about 3 h (Davis et al., 1991). Thus, the N/L ratio is unlikely to be affected by the sampling procedure itself, particularly if animals are trained on it. In rodents, elevated N/L ratios were associated with chronic stress exposure (Swan and Hickman, 2014; Hickman, 2017), and in monkeys, elevated N/L ratios were found during initial chair restrain training (Lee et al., 2013). The finding that in our data there was no difference between holiday and laboratory periods, and also no difference in comparison to baseline does not per se exclude the possibility that laboratory procedures would exert some acute stress. However, the finding does indicate that there is no lasting, cumulative effect, which is expected to show up in a change of the N/L ratio over time. Because the N/L ratio is also modulated by inflammatory responses (Forget et al., 2017), it additionally underlines the results on the absence of chronic inflammatory responses.

In conclusion, the analysis of various blood parameters as indicators for the severity of a variety of typical neuroscientific procedures, and their comparison to baseline parameters obtained before start of any laboratory work, did not provide evidence for cumulative effects resulting from the combination of procedures and it did not provide evidence for cumulative effects over time. Our data indicate that neuroscientific procedures have a much smaller influence on animal well-being and health than often assumed. The procedures and methods we investigated in this report vary only little across most NHP neurophysiology laboratories. Given overall beneficial conditions for the physiological and psychological well-being of animals, in terms of social housing in highly enriched environments, positive reinforcement techniques during husbandry, veterinary, and laboratory training, state-of-the-art experimental methods and well-educated staff at all levels, our data suggest that typical procedures required in neuroscience research do not exert a more than mild influence on the health and well-being of the animals.

## Synthesis

Reviewing Editor: Siegrid Löwel, University of Goettingen Decisions are customarily a result of the Reviewing Editor and the peer reviewers coming together and discussing their recommendations until a consensus is reached. When revisions are invited, a fact-based synthesis statement explaining their decision and outlining what is needed to prepare a revision will be listed below. The following reviewer(s) agreed to reveal their identity: Valeska Stephan.

The manuscript “Blood analysis of laboratory Macaca mulatta used for neuroscience research” provides an extensive investigation on whether and to what extent standard experimental procedures with non-human primates (NHPs), as widely used in systems neuroscience, can cause cumulative harm. For this, an extensive database of blood samples was analyzed which was taken over 15 years in a total of 39 rhesus macaques. These data analysis was supplemented with extensive ethological score sheets produced by a veterinarian regularly over many years. Despite sophisticated analysis, the study did not find any evidence for cumulative effects from fluid control, cranial implants, and laboratory work of the animals. This is a very welcome analysis in the field.

Both the topic and aim of the study are of great importance and interest in systems neuroscience, in particular for the community working with non-human primates (NHPs). Given the importance of the welfare of laboratory animals, specifically primates, finding objective, physiological indicators of the animals’ welfare and health status is needed to provide optimal care, specifically in relation to experimental and laboratory procedures.

While the topic is extremely important regarding the long term well-being of these animals, the publication in its current state has quite a number of shortcomings which need resolving to be qualitatively impactful. In general, the writing needs more care, precision and consistency, i.e. thorough editing.

Main points of critism:

- Preparation and description of the data

Generally, we find the actual data is not yet well enough prepared and described. We are missing some basic descriptive statistics (how many samples per animal, age, status of the animals, how long have they been in the laboratory, how many samples for each condition...) in addition to figure 1A (which only shows a subset of the data). In the same vain, we find the classification and description of the data sets for each of the analyses unclear and/or incomplete. (Providing the raw data would probably resolve some of the issues mentioned.)

Specifics:

Line 111: Blood samples are indexed but these indexes don’t play a role anymore in later analysis (especially “reason for sampling” and “anesthesia”)? Why index it when it’s not relevant? What subsets were drawn? What was the number of n for each subset? (see also Line 188).

Line 123: What was the reason for sampling outside surgery? What was the frequency of these samples?

Line 188: Earlier it was said that the samples were indexed (Line 111). Now samples are assigned to three groups (which relate to experimental status of the animal). So how is the data actually classified? Do the indexes play a role?

Line 197: The condition “holiday” seems to start on the 11th free day after a working period. Please describe the motivation behind this choice. Why 10 days? Any data on the 10 days in between? How is the time before and after surgery classified (I assume animals would have free access to water in this time)? Is data from this time included?

Line 241: Clarify what „all samples were drawn due to routine health checks” means. What kind of procedures were done as “routine health checks”. According to line 114 it seems a lot of samples were drawn during surgeries, which I would not classify as routine health checks since they were done in relation to surgery which is for experimental reasons and not veterinary health check.

Line 268: What is the n on the data set?

Line 287: Are these samples numbers from individual animal or from all animals? What is the number n altogether?

Line 295. Years with lower number of leukocytes in Fig. 2C are rather 9, 10, 11 (not: 8,10,11).

Line 320: Are the three groups identical to the ones described in Lines 188 ff.? If so, the additional information about classifying according to hydration status should be in the paragraph following Line 188.

Line 326: Generally, I don’t understand the format “N = x - y” to describe the sample size. Does it mean “between x and y samples”? Why not give a plain n? Also, these numbers for the Ns given don’t match with table 2. For example, in this line it say N = 17 - 26, when table 2 says the maximal N was 27 and the minimum was 5.

Line 326: Does the averaging procedure results in basically each monkey contributing one data point to each data set? So an N of 18 (Line 343) is equal of data from 17 animals?

Line 385: Do these groups correspond to the classification of baseline, holiday and laboratory (Line 188ff)? If so, this should be made clear (consistency of naming) or it should be clear these groups are classified differently.

Line 404: How can there be an N of 34 here, but for all blood samples the max N is 27 (table 2)? Why were animals not in laboratory procedures excluded since they could have provided a baseline?

Figure 1A/Line 243: What is the number of n? How many animals are represented? How was the subset chosen?

Figure 1B: What is the number of n?

- Description of methods

The balance between the detail in descriptions in the method section/about the methods is uneven. The description of the implant surgery seems very detailed (yet still lacking of certain information), while other methods of data collection are very superficial.

Specifics:

Line 130: Generally, on the one hand the description of the surgery procedures seems very detailed compared to descriptions of other methods. On the other hand, important information is still missing. So either the description should be extended to include the missing information and give a complete picture of the procedure, or it should be shortend to focus only on the relevant steps of the procedure (the ones which may lead low post-operative inflammation).

Line 137ff: It does not become clear what the difference is and therefore how this is a refinement. Given that using an acrylic headcap cannot be considered to be a refinement in terms of implant procedures in general, it has to become very clear and obvious why using this old technique is a refinement after all.

Line 140: Why is the cap implanted so early? And for what?

Line 159: What is the purpose of the opening?

Line 165: Is this the refinement referred to in Line 137?

Line 171: Why has the opening to be kept open?

Line 175: Is there an advantage using the horizontal primate chair instead of the upright primate chair?

Line 126: Generally, given that animal training is important in terms of creating a environment which is as stress-free as possible, more details on training and handling procedures would be good. How were the animals trained? What was the training regime? When were animals considered to comply willingly/stress-free?

Line 189 (also Line 567): More information on the animals status is needed: How were they acclimatized to the lab environment and their social groups? Age? What have they experienced yet? Have they experienced fluid deprivation yet (due to living in groups)? What is the handling situation? What are the overall handling and lab conditions? Who does the animal handling? How are these persons trained?

Line 371: Please describe the circumstances of when blood samples are taken more closely, especially in relation to what kind of stress animals could have experienced during sampling. Since the authors are interested in the effects of laboratory procedures it is important to ensure that the impact on parameters of measuring stress primarily come from those laboratory procedures and the impact of blood sampling is minized.

Line 202: Given that the behavioural assessment is analysed closely later, the description of the method of data collection is insufficient. Who did the scoring? How were they trained on it? How did the authors take care of bias in the scoring (inter- & intra observe bias)? How were the animals scored in general (In the cage? In the chair? In group? Alone?). Were all the attributes scored in one session (if so, how were the muscosal membrane scored while scoring social behaviour)? What is the frequency of scoring?

Line 205: Which scores were summed up here? What does “scores” refer to in your calculation?

Line 245 (and Figure 1). The unit of the red blood cell concentration (5.6 T/l) is uncommon in the biomedical world. Consider explaining, e.g., 1T = 10E12).

Confusion between percent (%) and permille (‰):

Line 424. 13/4835 is 0.27 % or 2.7 ‰ (not 0.027 ‰)

Line 425. 196/20,078 is 0.98 % or 9.8 ‰ (not 0.098 ‰)

Figure 2. Panel A. The labelling of the y-axis of the Leukocyte subfigure is unclear. Consider expanding to “Cell count (G/l)” Also, the unit for cell count (G/l) is unconventional. Consider explaining in the legend or changing to a more conventional unit, e.g., 10E9/l or similar.

Panel C. The top subfigure shows 11 histograms, but there are only 10 data points in the bottom subfigure (2-11 years after implantation).

Figure 3. The term ‘training’, as stated in the figure legend, is labelled ‘laboratory’ on the x-axis of Fig. 3A. Please adjust to either one in the figure or legend/text.

Figure 5A. The percentages in the small pie chart should be “%", not “‰” (permille):

‘+’ 193 / 24913 = 0.775 %

‘++’ 14 / 24913 = 0.056 %

‘+++’ 2 / 24913 = 0.008 %

Please correct!

Figure 5D. What does the y-axis in the inset graph show, fraction of all score sheets with score >0? In percent or permille? Please clarify!

- Aspects of the fluid schedule of the animals need to be clarified.

Specifics:

Line 196: What’s a generous amounts?

Line 314, Line 323, Line 524: Clarify use of the term ad libitum/ad lib. It is unclear if animals had ad lib access to fluids or not. Is “non-restricted” used interchangeably with ad libitum or is it something else?

Line 320: Given that animals were housed in small groups, how did you ensure animals belonging to the ‘baseline’ condition had ad libitum fluid and were not restricted? One draw back of the practicality of social housing can be that once even just one animal in the group is on a restricted schedule all other animals in the group usually may be effected by this and have restricted access to fluids as well to some degree. There are different strategies to circumvent this. How was it ensured that ad lib animals were truly ad lib and not “semi restricted”?

Line 323: Upon the time of sampling, when was the last time the laboratory group had access to fluid prior to sampling? This detail is to ensure that there may not be any short term effects of hydration on the samples (and on the indicators measured) and what is compared is indeed ad lib physiological states versus fluid-restricted physiological states.

- Various comments on data handling and analysis

Specifics

Line 199: The importance of body weight as indicator for health was pointed out. Hence it would be useful to provide a brief summary of the body weight of the animals.

Line 227: While using literature data as reference is certainly appropriate, it is unfortunate that the authors missed the chance to make use of the large data collection provided by the Primate Aging Database. It would have allowed for creating and analysing a more specifically tailored data set from animals in non-experimental settings as comparison.

Line 270: Please insert a Literature reference for the choice of parameter!

Line 294: Is the data on the number of leukocytes shown?

Line 318: Please insert literature reference for choice of parameters!

Line 370: Please describe the N/L parameter in more detail. Is it measuring short-term effects? Can it be used to measure long-term effects? Does anaesthesia have an effect on the parameters?

Line 437: Pointing out that the data considers data from up to 11 years seems irrelevant since most of the data was averaged and finer details and meta data, such as temporal information therefore is lost.

Line 528: It is indicated here that the animals in this study showed below-average uptake of water on Mondays and a steady average body weight, but no data is given to support this. (see also comment on Line 199)

Line 605: No data for primates available?
